# Refractory immune cytopenia successfully treated with mycophenolate mofetil in four adolescents with del22q11.2 syndrome

**DOI:** 10.3389/fimmu.2026.1819182

**Published:** 2026-05-13

**Authors:** Cristina Cifaldi, Lucia Pacillo, Chiara Rossetti, Silvia Di Cesare, Michele La Manna, Veronica Santilli, Beatrice Rivalta, Elisabetta Lembo, Mattia Moratti, Lucia Colucci, Gigliola Di Matteo, Paolo Palma, Federica Pulvirenti, Emma Concetta Manno, Giuseppe Palumbo, Donato Amodio, Caterina Cancrini

**Affiliations:** 1 Department of Systems Medicine, University of Rome Tor Vergata, Rome, Italy; 2 Research Unit of Primary Immunodeficiencies, Bambino Gesù Children’s Hospital, IRCCS, Rome, Italy; 3 Clinical Immunology and Vaccinology Unit, Bambino Gesù Children’s Hospital, IRCCS, Rome, Italy; 4 Department of Pediatric Hemato-Oncology and Cell and Gene Therapy, Bambino Gesù Children’s Hospital, Scientific Institute for Research and Healthcare (IRCCS), Rome, Italy; 5 Residency School of Pediatrics, University of Rome “Tor Vergata”, Rome, Italy; 6 Pediatric Unit, IRCCS Azienda Ospedaliero-Universitaria di Bologna, University of Bologna, Bologna, Italy; 7 PhD Program in Immunology, Molecular Medicine and Applied Biotechnology, University of Rome Tor Vergata, Rome, Italy; 8 Reference Centre for Primary Immune Deficiencies, Sapienza University Hospital Policlinico Umberto I, Rome, Italy

**Keywords:** de22q11.2 syndrome, DiGeorge syndrome, immune thrombocytopenia, mycophenolate mofetil, targeted therapy

## Abstract

**Introduction:**

Chromosome 22q11.2 deletion syndrome (22q11DS) presents a wide variability of phenotypic features, including different grades of immune dysfunctions, leading to increased susceptibility to infections, autoimmune diseases and atopy. The most common autoimmune manifestation in 22q11DS patients is immune thrombocytopenia (ITP), which is often relapsing and refractory to standard therapy.

**Methods:**

We present a cohort of four pediatric/adolescents 22q11DS patients presenting with refractory ITP, treated with low dosage Mycophenolate Mofetil (MMF) for more than 24 months. We performed complete deep longitudinal immunological investigations by multiparametric flow cytometry, and monitored blood counts as well as EBV viremia.

**Results:**

All four patients didn’t experience any relapse since the beginning of MMF therapy. Three out of four showed a complete remission with PLT > 100000/uL. All the patients presented immunological features reported to be associated with immunodysregulation: reduced naïve CD4+T cells and memory B cells, increased cTfh cells and reduced Treg cells frequency. During MMF treatment we detected a decrease in cTfh frequency and a reduction in PD-1 expression along with a moderate recovery of regulatory T cells.

**Discussion:**

MMF treatment was associated with sustained platelet stabilization in this cohort. Specific immunological biomarkers could help monitor treatment response, guide clinicians in selecting targeted therapies and may be used to monitor the response to therapy over time.

## 1 Introduction

Chromosome 22q11.2 deletion syndrome (22q11DS) is the most common microdeletion disease in humans, with a prevalence of 1:4000 to 1:6000. There is wide variability of phenotypic features that can include cardiac anomalies, hypoparathyroidism, thymic hypoplasia or aplasia, palatal defects, feeding and swallowing abnormalities, neuropsychiatric disorders, renal anomalies, and others. Up to 75–80% of 22q11.2DS patients exhibit derangements of the immune system, which can lead to increased susceptibility to infections, autoimmunity, and atopy (1, 2). The most common autoimmune manifestations in 22q11.2DS patients are autoimmune cytopenia (AIC), in particular immune thrombocytopenia (ITP), autoimmune thyroiditis and juvenile idiopathic arthritis. AIC in 22q11.2DS patients are often difficult to manage, resulting in relapsing and refractory to first line therapies (2). Defective thymic differentiation, impaired Treg cells function and/or altered peripheral T cell homeostatic proliferation with increased response to self-antigens could contribute to immunodysregulation phenomena facilitated often by persistent viral infections, especially chronic Epstein-Barr-Virus (EBV) infection (2). In an Italian cohort of 22q11.2DS patients, those with AIC presented common immunological features, including decreased percentage of CD4 naïve T cells and recent thymic emigrants (RTE), decreased switched memory B cells and increased CD21low B cells compared to those without AIC (3). Moreover, in 22q11.2DS patients Treg cells have been reported to be often decreased, contributing to autoimmune manifestations (4). According to national and international guidelines for AIC treatment (5–10), first line therapies for ITP include high dose intravenous immunoglobulins (IVIg), while second line therapies encompass steroids, mycophenolate mofetil (MMF), sirolimus, TPOR agonists (eltrombopag, romiplostim) and Rituximab (11). Mycophenolate mofetil is a prodrug of mycophenolic acid (MPA), an inhibitor of inosine-5’-monophosphate dehydrogenase (IMPDH). MPA depletes guanosine nucleotides preferentially in T and B-lymphocytes and inhibits their proliferation, thereby suppressing cell-mediated immune responses and antibody formation. Targeting both T and B autoreactive cells, it is used as second-line therapy in refractory AIC, off-label for indication. In a single-center experience, Miano et al. (12) reported that 65% (22/34) of children with AIC refractory to first line treatment showed a good response to MMF, especially in ALPS patients (11/11, 100%). This finding was confirmed in another single-center study in the USA (13) reporting an excellent response (12/13) to MMF in ALPS patients with refractory autoimmune cytopenia. Herein we present a cohort of four pediatric/adolescents 22q11DS patients presenting with refractory ITP, treated with low dosage MMF for more than 24 months.

## 2 Methods

### 2.1 Ethics and informed consent

All procedures performed in the study were in accordance with the ethical standards of the institutional research committee and with the 1964 Declaration of Helsinki and its later amendments. Informed consent, following standard ethical procedures, was obtained from the case-index patient and her parents. This study was approved by the Institutional Ethical Committee of Bambino Gesù Children Hospital (Prot. n. 914; date of approval: 3 November 2023). All subjects gave their informed consent to perform analysis. The studies were performed at the Bambino Gesù Children Hospital Rome.

### 2.2 ITP response definitions and clinical endpoints

ITP Response Definitions and Clinical Endpoints Clinical response and outcomes were defined according to the International Working Group (IWG) consensus criteria. Complete Response (CR) was defined as a platelet count ≥ 100 × 10^9^/L and the absence of bleeding. Response (R) was defined as a platelet count ≥ 30 × 10^9^/L with at least a 2-fold increase from the baseline count and the absence of bleeding. No Response (NR) was defined as a platelet count < 30 × 10^9^/L, less than a 2-fold increase from baseline, or the presence of bleeding (14). Sustained response was defined as > 3 of 4 platelet counts > 50 x 10^9^/L during weeks 6–12 without rescue therapy. The baseline platelet count was established as the value recorded immediately before the initiation of Mycophenolate Mofetil (MMF) therapy. Loss of response (relapse) was defined as a platelet count falling below the thresholds for R or CR or the recurrence of clinical bleeding. Time to response was measured from the start of MMF treatment to the first achievement of CR or R. Clinical safety and meaningfulness were further assessed by the absence of major bleeding events, the discontinuation of rescue therapies (such as corticosteroids or high-dose IVIg), and the avoidance of hospitalization or splenectomy. Given the underlying immune condition of 22q11.2DS and the pediatric/adolescent nature of the cohort, patients were considered refractory based on their lack of sustained response to multiple medical lines of therapy rather than the criteria involving splenectomy.

### 2.3 Peripheral blood immunophenotype

The analysis was carried out on EDTA peripheral blood samples and processed within 24 hours of venipuncture. Erythrocytes were removed using ammonium chloride and lymphocytes were washed, resuspended in phosphate-buffered saline (PBS; Sigma-Aldrich), and stained with the following mouse anti-human monoclonal antibodies: CD45RA (clone T6D11), CD3 (clone BW264/56), CD4 (clone REA263), CD27 (clone M-T271), TCR αβ (clone T10B9), TCR γδ (clone 11F2), CD8 (clone REA734/HIT8a), CCR7 (clone 3D12; Thermo Fisher Scientific Inc., MA, USA), and CD19 (clone REA675/SJ25-C1) (Miltenyi Biotec, Bergisch Gladbach, Germany); CD16 (clone 3G8), CD56 (clone MY31), CD24 (clone ML5), CD21 (clone B-ly4), IgD (clone IA6-2), goat F(ab’)_2_ anti-human IgM (μ chain) (Jackson ImmunoResearch, Cambridge House, UK) and CD38 (clone HIT2) (BD Biosciences, San Jose, CA, USA).

### 2.4 Circulating T follicular helper and peripheral regulatory T cells immunophenotype

Circulating T follicular helper (cTfh) cells and related subsets were analyzed in freshly collected whole blood samples. Cells were stained with monoclonal antibodies specific for CD4 (clone REA263), CD45RA (clone T6D11), CXCR5 (clone REA102/RF882), CXCR3 (clone REA232/GO25H7) and PD-1 (clone REA1165) all from Miltenyi Biotec, Bergisch Gladbach, Germany. Following staining, erythrocytes were removed using BD FACS Lysing Solution, and leukocytes were washed with PBS (Sigma-Aldrich).

Isolated Peripheral Blood Mononuclear Cells (PBMC) were pre-treated with Fc-blocking reagent (Thermofisher Scientific Inc., MA, USA), then surface stained, for 30 min at room temperature, to identify regulatory T-cell (Treg) using the following antibodies: CD4 (clone REA263; Miltenyi Biotec, Bergisch Gladbach, Germany), CD45RA (clone T6D11; Miltenyi Biotec, Bergisch Gladbach, Germany) or CD45RO (clone UCHL1 BD, Pharmigen), CD127 (ebioRDR5, Thermofisher Scientific, Inc., MA,USA), CD25(clone M-A251, BD Biosciences, San Jose, CA, USA), for the membrane staining and FoxP3 (clone PHC101, Thermo Fisher Scientific Inc., MA, USA) and Helios (clone 22F6, BioLegend San Diego, California, USA) for transcription factors. Intracellular staining was performed with Foxp3 Transcription Factor Staining Buffer Set (Thermo Fisher Scientific Inc., MA, USA) according to the manufacturer’s instructions. At least 200,000 CD4+ events within lymphocyte gate were acquired for each sample.

Data acquisition was performed on a FACSCanto II flow cytometer (BD Biosciences, San Jose, CA, USA) and FlowJo software (Tree Star Inc., version 9.3.2) was used for all analyses. The analyzed cell populations, together with the corresponding marker combinations used for their identification and relative gating strategies, are detailed in **Supplementary Table 1**, **Supplementary Figures 1-3**.

### 2.5 EBV viral load monitoring

EBV viral load was longitudinally monitored by quantitative PCR on whole blood samples during follow-up and expressed as copies/mL. Sampling frequency followed routine clinical monitoring during follow-up visits and immunophenotyping timepoints.

### 2.6 MMF dosage

MMF is approved for immunosuppression in kidney transplanted patients in the dosage of 1200 mg/m2/day in two doses. Since immunosuppression increases susceptibility to bacterial, viral and fungal infections and the risk of development of lymphoma and other malignancies, it is mandatory to choose the minimum dose required to guarantee a good blood cell count and avoid the adverse events of chronic immunosuppression especially in PID patients. For this reason, we used a medium dosage of 375 mg/m2/day (range 264–433 mg/m2/day) with the intent to titrate it based on clinical response and drug tolerability. Treatment adherence was evaluated during routine clinical follow-up through review of pharmacy refill records and structured patient interviews.

### 2.7 Statistical analyses

Data are presented as median (interquartile range, IQR). Statistical comparisons between groups were performed using the Mann–Whitney U test. Statistical significance is indicated with asterisks (*, P < 0.05; **, P < 0.01; ***, P < 0.001; ****, P < 0.0001). Statistical analyses were conducted in GraphPad Prism 10.

## 3 Results

### 3.1 Del22q11 cohort characteristics

We present a cohort of four adolescents and young adult patients (median age: 13.1 years; male:female ratio 2:2) affected by DiGeorge Syndrome (DGS), who experienced recurrent and refractory AIC.

Pt-1 is a 21-year-old Italian girl who was diagnosed with 22q11DS at 8 months of age, due to neurodevelopmental and speech delay and typical facial features. Her clinical history was characterized by several autoimmune and neoplastic manifestations. She was diagnosed with autoimmune thyroiditis at 3 years of age; juvenile idiopathic arthritis at 5 and papillary thyroid carcinoma at 10. Moreover, she experienced recurrent respiratory infections since the first year of life and chronic EBV viremia. She presented the first episode of ITP (54000/uL) at 13 years of age, with recurrent relapses, treated with high dose IVIg and Rituximab without remission. She was eventually started on MMF at a dosage of 426 mg/m^2^/die. She also started ScIg replacement therapy. Since the beginning of MMF therapy, no clinical relapses were detected and MMF dosage was progressively reduced and ultimately discontinued, with platelet counts remaining stably above 100000/µL.

Pt-2 is an Italian 16-year-old boy who was diagnosed with 22q11DS at birth due to neonatal hypocalcemia, typical facial features and tetralogy of Fallot cardiopathy. His clinical history was characterized by congenital kidney malformation, complex genital anomaly surgically corrected, palatal defect, neurodevelopmental delay, rhinolalia, mild hearing defect, speech delay, recurrent respiratory infection, asthma and vitiligo. He presented the first episode of ITP (6000/uL) at 3 years of age, treated with a high dose of IVIg with good response. Platelet count remained stable among 90000 and 120000/uL till the age of 11 years, when he developed ITP with recurrence of one episode every month, requiring monthly high-dose IVIg. Chronic EBV viremia and splenomegaly were detected. He also experienced autoimmune neutropenia starting at age of 12. He was started on MMF at a dosage of 378 mg/m^2^/die. Since the beginning of MMF therapy, no clinical relapses were detected and MMF dosage was progressively reduced till its interruption after 24 months of therapy. No relapses were recorded during the two-year follow-up after MMF interruption.

Pt-3 came to our attention at the age of 8. She is an 18 years-old girl from Ukraine affected by neurodevelopmental and speech delay and typical facial features since birth. Her clinical history was characterized by atopic dermatitis and recurrent infections. Chronic EBV viremia was detected. She presented Evans syndrome, AIHA and ITP (10.3 g/dL and 108000/uL), at 6 years of age, treated with steroids. She was evaluated in Italy at 8 years of age for suspected myelodysplasia that was excluded but she was eventually diagnosed with 22q11DS. When she moved to Italy at 13 years of age, MMF was stopped and she presented with an ITP relapse (95000/uL). Then she was started on MMF at a dosage of 433 mg/m2/die showing no clinical relapses and MMF dosage was progressively reduced till the minimum dosage (62.5 mg/die) to maintain stable platelets count > 100000/uL.

Pt-4 is a 19 years-old boy with mixed origin (Italian-Saint Domingo) who was diagnosed with 22q11DS at birth due to neonatal hypocalcemia and typical facial features. His clinical history was characterized by neurodevelopmental delay and behavioral psychiatric problems. Chronic EBV viremia and splenomegaly were detected. He presented the first episode of ITP (72000/uL) at 7 years of age, treated with steroid therapy. He presented relapsing refractory ITP, requiring high dose IVIg, high dose steroids and Rituximab, with poor response. He also presented an episode of autoimmune neutropenia. He was started on very low dosage MMF (264 mg/m^2^/die) with poor compliance, in association with IVIg replacement therapy. He is currently receiving 2g/die of MMF with ITP relapse although reduced in frequency.

### 3.2 Immunological features

**Figure 1:**
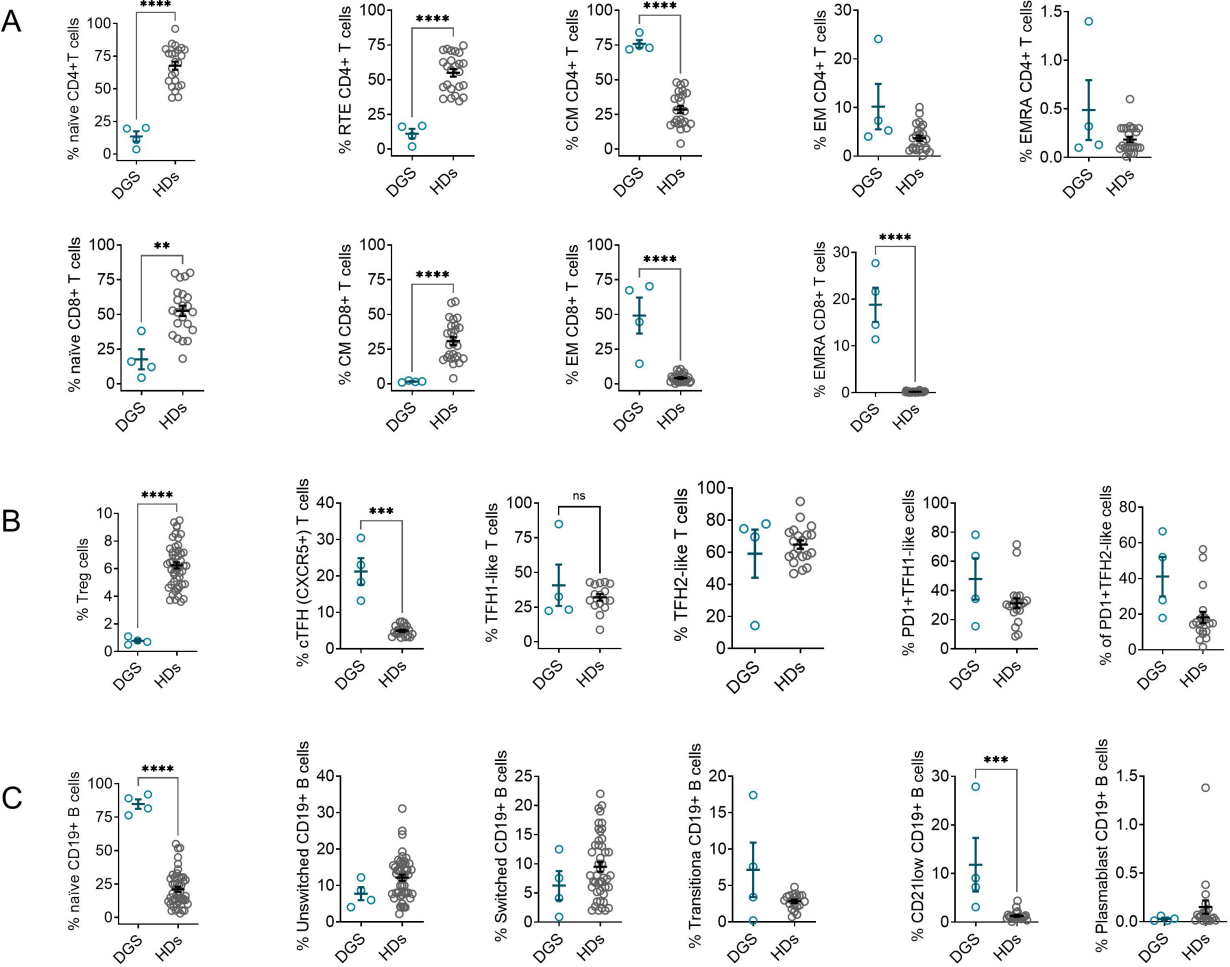
Immunological features in patients with 22q11.2 deletion syndrome. Frequencies of peripheral blood T- and B-cell subsets in patients with DGS (DGS, n = 4) compared with age-matched healthy donors (HDs, n = 23). **(A)** T- cell subset **(B)** T regulatory cell and T follicular helper and **(C)** B-cells subsets. Data are shown as median (interquartile range, IQR). Statistical comparisons between groups were performed using the Mann–Whitney U test. Significance levels are indicated as **p < 0.01, ***p < 0.001, ****p < 0.0001; ns, not significant.

At baseline, all patients showed features consistent with immune dysregulation. CD4^+^ T cells demonstrated a similar profile, including a marked reduction in naïve CD27^+^CD45RA^+^ cells and recent thymic emigrants (RTEs; CD31^+^CD45RA^+^), and concomitantly increased frequencies of central memory (CM; CD27^+^CD45RA^-^) and effector memory (EM; CD27^-^CD45RA^-^) subsets. CD8^+^ T cells exhibited a more severe imbalance, with reduced naïve and CM subsets and a corresponding expansion of EM and EMRA populations, suggesting a shift toward terminal differentiation (**Figure 1A**). T regulatory cells (Tregs; CD4^+^CD25^+^CD127^lowFoxP3^high) were significantly decreased. Circulating T follicular helper cells (cTfh; CD4^+^CXCR5^+^) were markedly increased and showed elevated PD-1 expression, indicative of chronic activation and immunosenescence (**Figure 1B**). In the B cell compartment, we observed an expansion of naïve B cells (CD27^-^IgD^+^IgM^+^), a reduction in switched memory B cells (CD27^+^IgD^-^IgM^-^), and an increase in transitional B cells (CD38^++^IgM^++^). CD21^lowCD38^-^ B cells were also significantly expanded (**Figure 1C**). Patients with greater CD21^low B cell expansion exhibited a stronger Th1-like polarization. This immunophenotype is consistent with patterns described in systemic lupus erythematosus (SLE), rheumatoid arthritis, Sjögren’s syndrome, and previously reported DGS cases (3, 14).

### 3.3 Normalization of platelets after MMF

**Figure 2:**
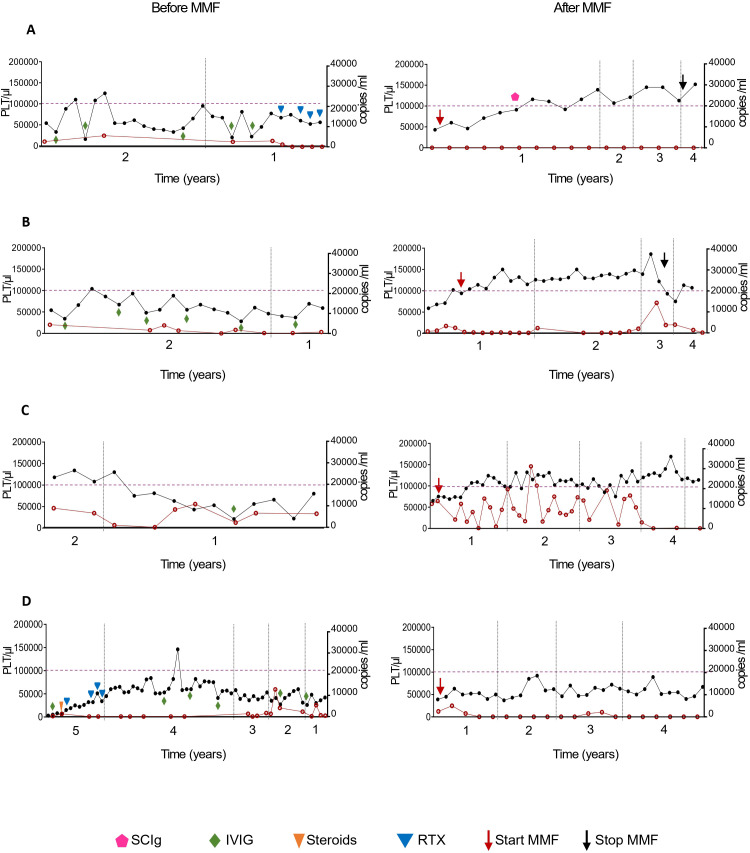
Longitudinal evaluation of platelets.Right and left graphs represent years before and after MMF therapy follow-up, with normalization of platelet counts (black) in three of the four patients during the follow-up. Longitudinal platelet counts and EBV viral load before and after MMF treatment in patients with 22q11.2 deletion syndrome. Longitudinal evaluation of platelet counts (PLT, black dots; left y-axis) and EBV, Epstein–Barr virus viral load (red dots; right y-axis) in patients with 22q11.2 deletion syndrome (22q11.2DS) before and after initiation of mycophenolate mofetil. **(A)** Patient 1 (pt-1); **(B)** Patient 2 (pt-2); **(C)** Patient 3 (pt-3); **(D)** Patient 4 (pt-4). For each patient, the left panels show platelet trends before MMF initiation (Before MMF), whereas the right panels show platelet counts during follow-up after treatment initiation (After MMF). The dashed horizontal line indicates the threshold for platelet count normalization (≥100,000/µL). Red arrows indicate MMF initiation, and black arrows indicate MMF discontinuation. Vertical dotted lines indicate relevant time points. Concomitant treatments are indicated as follows: intravenous immunoglobulins (IVIg, green diamond), corticosteroids (orange triangle) and rituximab (RTX, blue triangle).

We evaluated longitudinal immunological and clinical data from 22q11DS patients, focusing on the effects of MMF therapy and associated immune profile variations over time. Two of the four patients achieved normalization of platelet counts during follow-up (≥100,000/μl) with sustained remission for more than four years on therapy. Two patients discontinued MMF after 2 and 3 years and remained relapse-free post-therapy follow-up. One patient with low adherence (Pt-4) showed only partial improvement, with stable platelet counts around 50,000/μl. Treatment adherence was assessed through pharmacy refill records and structured patient interviews and was considered suboptimal in this patient, which may have contributed to the partial clinical response despite subsequent MMF dose escalation. No relapses were observed in any of the patients. Two patients had concurrent autoimmune neutropenia, which resolved spontaneously. Chronic Epstein–Barr virus (EBV) infection was present in all patients and remained stable throughout follow-up (**Figure 2**). No increase in infection frequency or occurrence of opportunistic infections was observed during MMF treatment. None of the patients received Pneumocystis jirovecii or antiviral prophylaxis. Immunoglobulin therapy was administered in two patients (SCIg in Pt-1 and IVIg in Pt-4). In Pt-1, SCIg was introduced as supportive therapy to reduce relapses of infection-triggered cytopenia. In Pt-4, IVIg was administered as an immunomodulatory treatment for refractory immune thrombocytopenia in the setting of suboptimal response to MMF and was already ongoing before MMF initiation. Mean immunoglobulin levels (IgG, IgA, IgM) before and after MMF treatment are reported in **Supplementary Table 2**.

### 3.4 Immunological features after MMF

**Figure 3:**
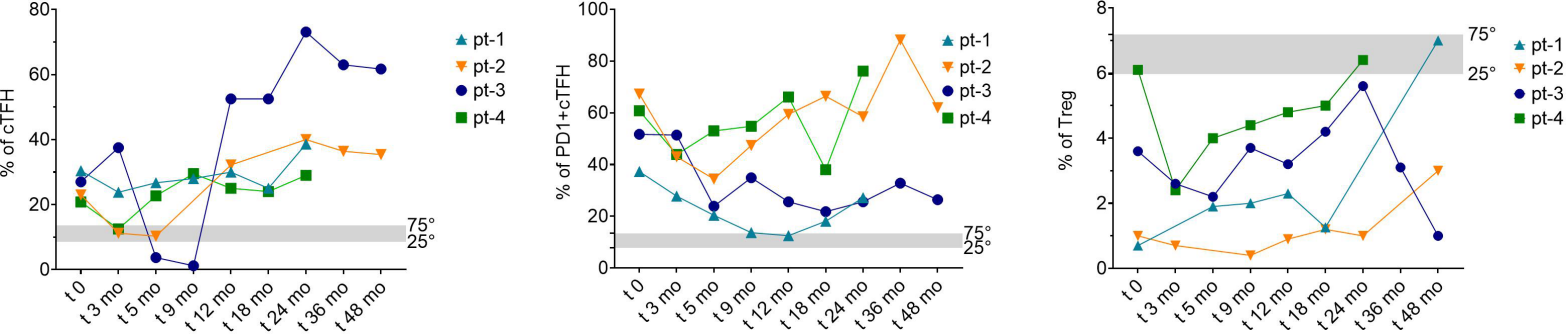
Longitudinal changes in circulating T follicular helper cells and regulatory T cells during MMF treatment in patients with 22q11.2 deletion syndrome. Longitudinal frequencies of cTfh, circulating T follicular helper cells, PD-1^+^ cTfh cells, and Tregs, regulatory T cells measured at baseline (t0) and during follow-up after initiation of MMF, mycophenolate mofetil therapy in four patients with 22q11.2 deletion syndrome (Pt-1 to Pt-4). Left panel: percentage of cTfh cells (CD4^+^CD45RA^-^CXCR5^+^) over time. Middle panel: percentage of PD-1–expressing cTfh cells. Right panel: percentage of regulatory T cells (CD4^+^CD25^highCD127^lowFOXP3^+^). The grey shaded areas indicate the interquartile reference range (25th–75th percentile) derived from age-matched healthy donors. Timepoints are expressed as months from MMF initiation.

Longitudinal immunophenotypic analyses up to 48 months post-MMF initiation revealed a consistent decrease in cTfh cell frequency along with a decrease in PD-1 expression on cTfh cells during the first months indicating a reduction in their senescent/exhausted phenotype. Moreover, we observed a progressive increase of Treg cells frequency during the early treatment phase, with trends maintained over time in three out of four patients. Pt-3 showed a transient increase in cTfh frequency following SARS-CoV-2 infection between months 5 and 9 (**Figure 3**), indicating a reappearance of immunological abnormalities during infectious episodes and suggesting an underlying fragility of T-cell homeostasis. Notably, in pt-3 an EBV reactivation was temporally associated with a marked decrease in Treg frequency and a concomitant increase in cTfh cells, whereas PD-1 expression did not show relevant fluctuations. Similarly, in pt-2 an EBV reactivation approximately five months after MMF initiation was followed by a subsequent increase in cTfh frequency and PD-1 expression without a parallel reduction in Treg cells. Overall, no consistent association between EBV fluctuations and platelet count variations was observed across patients. The CD4^+^ T cell compartment, frequencies of naïve and B cell maturation profiling improved over follow-up. CD21^low B cell frequencies decreased slightly in three of the four patients. Representative flow cytometry plots illustrating changes in cTfh cells, Tregs, switched memory B cells, and naïve T- and B-cell compartments before and after MMF treatment are provided in **Supplementary Figures 4-7**. Detailed longitudinal immunophenotypic changes before and after MMF treatment for each patient are provided in **Supplementary Figures 8–11**.

## 4 Discussion

Autoimmune cytopenias (AIC), particularly immune thrombocytopenia (ITP), in patients with 22q11.2 deletion syndrome (22q11.2DS) are clinically challenging due to frequent resistance to first-line therapies, including corticosteroids and IVIg (15). Rituximab, although widely used as a second-line agent, demonstrates inconsistent efficacy and poses risks of prolonged hypogammaglobulinemia and impaired B-cell reconstitution in this population (15). The persistent immune dysregulation observed in 22q11.2DS, aligns this syndrome with the broader category of combined immunodeficiencies (CID) presenting with cytopenia (16, 17). These disorders exhibit a distinct immunological profile, including reduced naïve T cells and defects in both regulatory T-cell (Treg) and T follicular helper (Tfh) cell compartments. Notably, the Tfh compartment is increasingly recognized as a central driver of immune dysregulation in primary immunodeficiencies and autoimmune diseases. Indeed, Tfh cell expansion has been documented in several primary immunodeficiencies (PID), such as CID, common variable immunodeficiency (CVID), autoimmune lymphoproliferative syndrome (ALPS), and in systemic autoimmune diseases including systemic lupus erythematosus (SLE), rheumatoid arthritis, and Sjögren’s syndrome (18–22). In 22q11.2DS, increased frequencies of circulating Tfh cells have been reported, and it has been suggested that this is correlated with autoimmune manifestations (2, 3, 16, 17, 19, 23).

Consistent with these observations, our patients exhibit a profound imbalance in the T-cell compartment, including a marked reduction in naïve CD4+ T cells and recent thymic emigrants reflecting the underlying thymic defect, alongside a shift in CD8+ cells toward terminal differentiation (EMRA) (3, 17). The concomitant reduction in Tregs likely compromises immune tolerance, while the marked expansion of activated cTfh cells at baseline, characterized by high PD-1 expression, a marker of chronic activation and immunosenescence (24), reflects a disordered adaptive response under peripheral homeostatic pressure (15, 17, 19). This immunological signature reinforces what was already described by Montin et al., who identified reduced naïve CD4^+^ T cells and switched memory B cells as strong predictors of autoimmune cytopenia development in 22q11.2DS (3). Collectively, these data suggest that Tfh and Treg compartments may serve as dynamic biomarkers of immune dysregulation.

Interestingly, similar immunological features have been described in immune-mediated cytopenias developing after hematopoietic stem cell transplantation (HSCT) for pediatric non-malignant disorders, where impaired thymic output, defective regulatory networks, expansion of activated follicular helper T cells, and persistent viral stimulation contribute to immune tolerance breakdown during immune reconstitution. In this context, 22q11.2DS may represent a congenital model of chronic immune reconstitution imbalance, supporting the hypothesis that therapeutic strategies targeting T-cell activation and Tfh–B cell interactions, including MMF, could have relevance across both congenital and acquired settings characterized by impaired thymic function and immune dysregulation (25).

In this context, our study demonstrates the clinical efficacy of long-term, low-dose MMF treatment in a cohort of pediatric and adolescent 22q11.2DS patients with refractory ITP. All patients experienced platelet stabilization following MMF initiation, with three achieving complete remission and one maintaining partial response in the setting of poor adherence. Importantly, two patients successfully stopped MMF and remained relapse-free throughout the follow-up period, supporting the durability of MMF-induced immune control. These findings are consistent with previous reports describing MMF as an effective therapeutic option in refractory autoimmune cytopenias associated with immune regulatory disorders, including ALPS and CID-like phenotypes (12, 13, 16).

Our longitudinal analysis following MMF therapy provides additional immunophenotypic observations that may offer indirect insight into mechanisms associated with treatment response; the rapid normalization of platelet counts in our cohort correlated with a decrease in cTfh frequency and a reduction in PD-1 expression during the first months. This suggests that targeted immunomodulation was associated with a reduction in PD-1 expression on cTfh cells, suggesting a possible shift away from a chronically activated phenotype (19). The progressive contraction of cTfh cells together with reduced PD-1 expression suggests a shift away from chronic activation and dysfunctional B-cell help, potentially reducing autoreactive germinal center activity, although functional studies would be required to confirm this mechanism. These findings are consistent with recent transcriptomic evidence showing that patients with 22q11.2DS exhibit expansion of follicular helper T cells with senescence-associated signatures, particularly in the setting of recurrent infections, together with impaired B-cell maturation and reduced class-switched memory B cells (26). This rebalancing, paralleled by moderate Treg recovery, and decreased Tfh frequencies could reflect modulation of Tfh–B cell interactions rather than broad immunosuppression. Indeed, similar immunophenotypic alterations involving regulatory T-cell compartments have also been reported in patients with Evans syndrome, in whom sirolimus treatment was associated with improvement of T-cell homeostasis and regulatory T-cell profiles, supporting the concept that targeted modulation of T-cell subsets may represent a shared feature across immune dysregulation disorders (27, 28).

Notably, a transient increase in cTfh cells was observed in one patient in association with the occurrence of a viral infection, supporting the notion that Tfh dynamics are sensitive to infectious and inflammatory triggers and may fluctuate in response to immune challenges. cTfh dynamics may represent candidate biomarkers associated with treatment response and could be explored in larger studies in 22q11.2DS-associated autoimmune cytopenia. In addition, patient-specific temporal associations between EBV reactivation episodes and fluctuations in cTfh and Treg compartments were observed in two patients as well as during SARS-CoV-2 infection, suggesting that persistent viral stimulation may variably influence immune regulatory dynamics in 22q11.2DS.

As expected, naïve T-cell frequencies did not recover during MMF treatment, probably due to the altered thymic function. T-cell–mediated immune dysregulation supports the use of MMF over B-cell–depleting therapies such as rituximab in 22q11.2DS patients. Rituximab, while effective in selected cases, carries the risk of prolonged hypogammaglobulinemia and impaired B-cell reconstitution in a population without a clear humoral defect (15). However, we cannot exclude that prophylaxis with Ig could play a role in decreasing the infection triggers. Therefore, MMF may contribute to modulation of T-cell activation and Tfh–B cell interactions, although this interpretation remains speculative and requires confirmation in mechanistic studies in CID-like immune dysregulation.

Such findings emphasize the importance of early, extensive immunophenotyping to guide personalized therapeutic approaches in 22q11.2DS patients presenting with refractory immune dysregulation. However, given the limited sample size, larger prospective studies will be required to confirm these associations and to better define their potential role as biomarkers of prognostic markers of immune complications and treatment response.

## Data Availability

The raw data supporting the conclusions of this article will be made available by the authors, without undue reservation.
