# Supplementary figures and images for: Refractory immune cytopenia successfully treated with mycophenolate mofetil in four adolescents with del22q11.2 syndrome
Source: Front Immunol. 2026 May 13;17:1819182. doi: 10.3389/fimmu.2026.1819182 (PMC13212233; doi:10.3389/fimmu.2026.1819182)

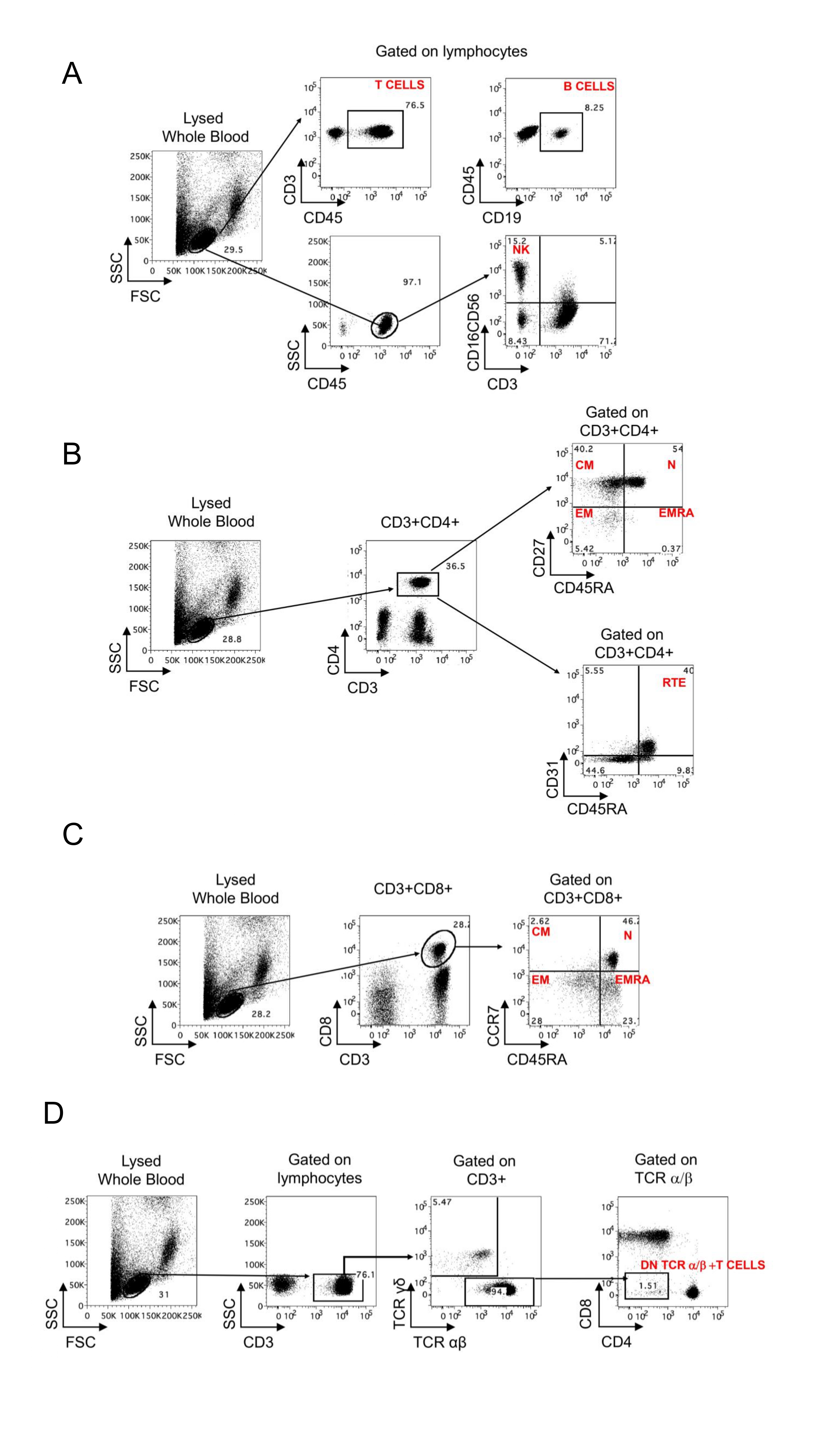

Supplement: Supplementary file 1 [file Image1.jpg]

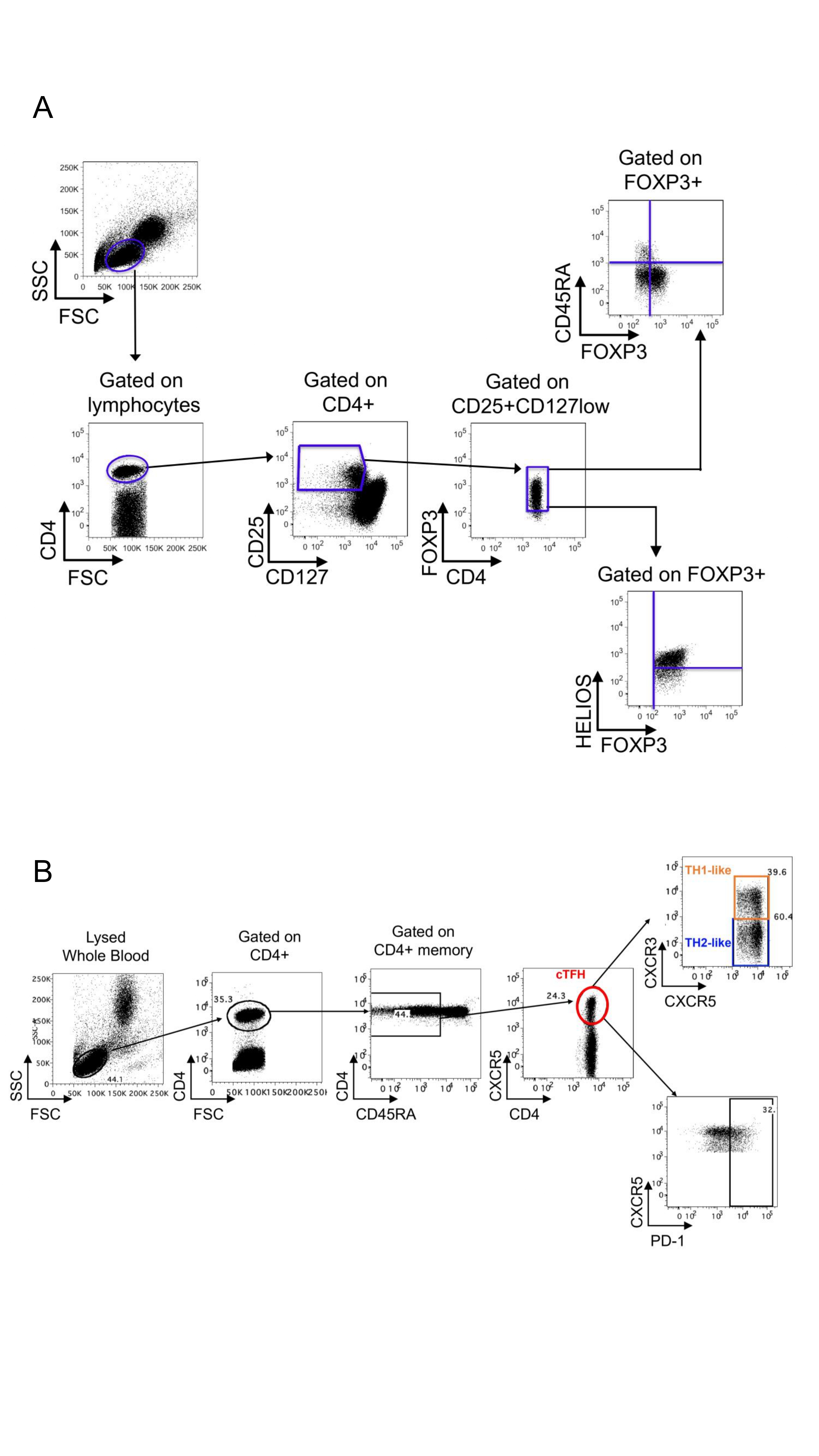

Supplement: Supplementary file 2 [file Image2.jpg]

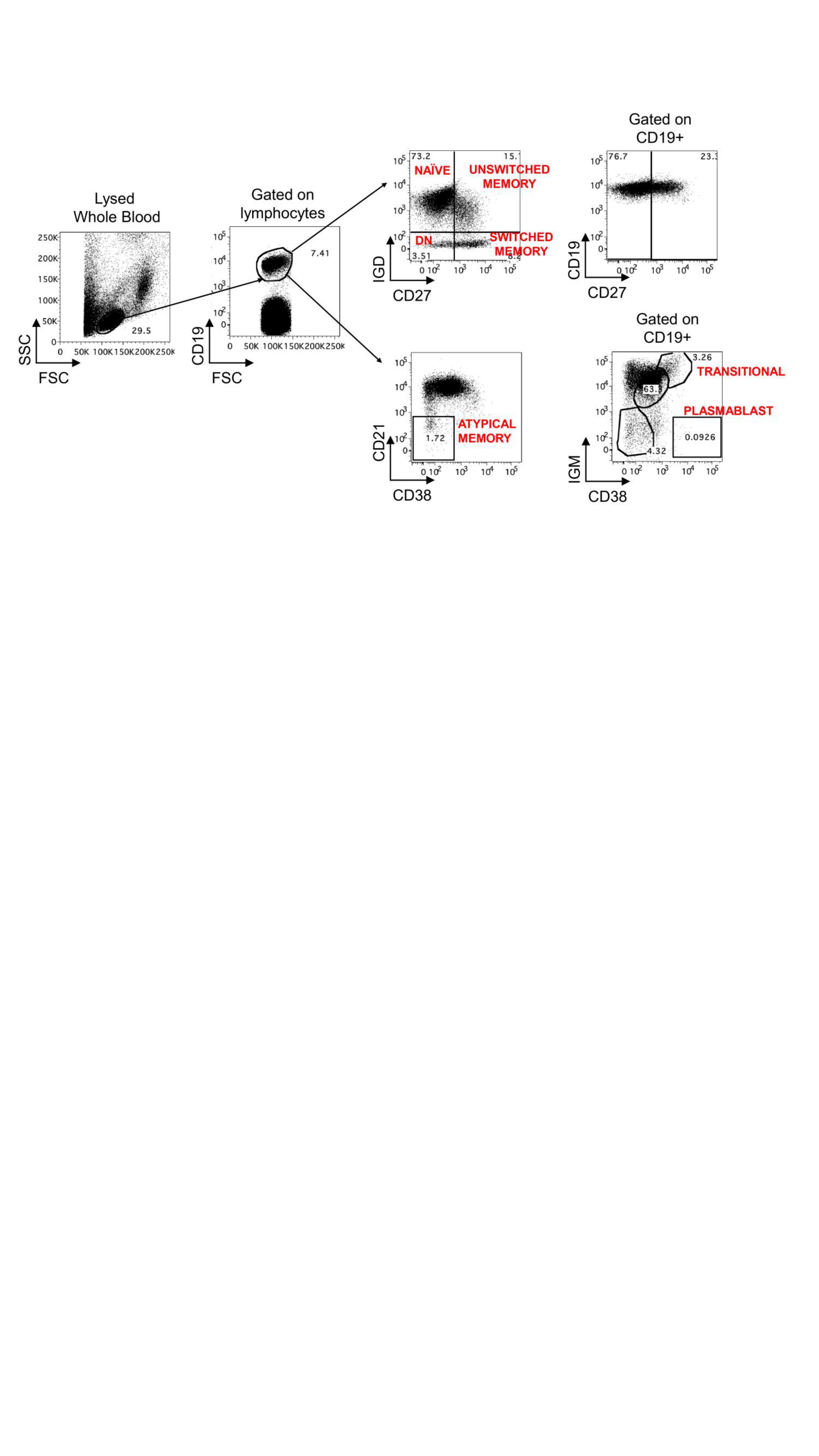

Supplement: Supplementary file 3 [file Image3.jpg]

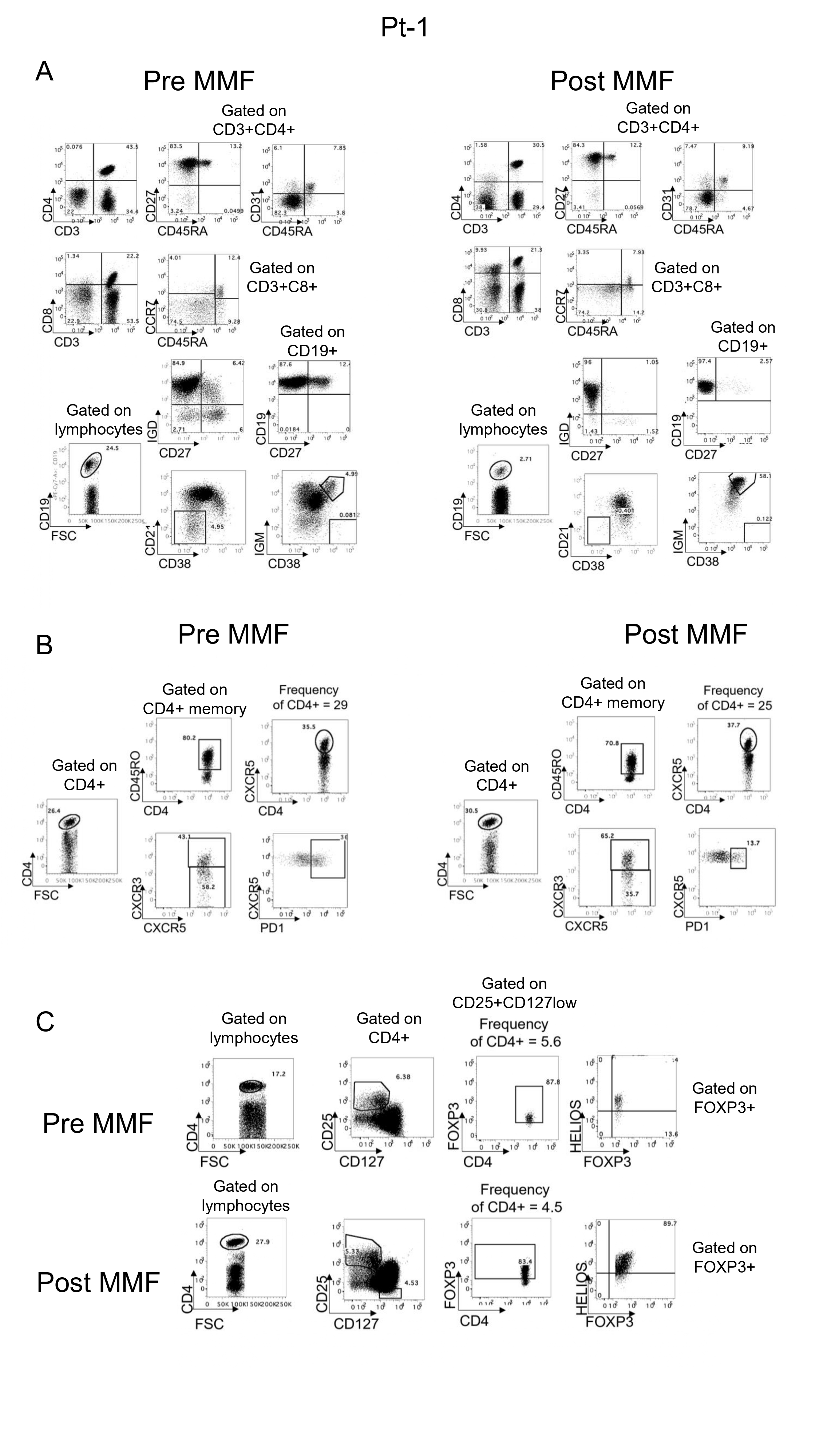

Supplement: Supplementary file 4 [file Image4.jpg]

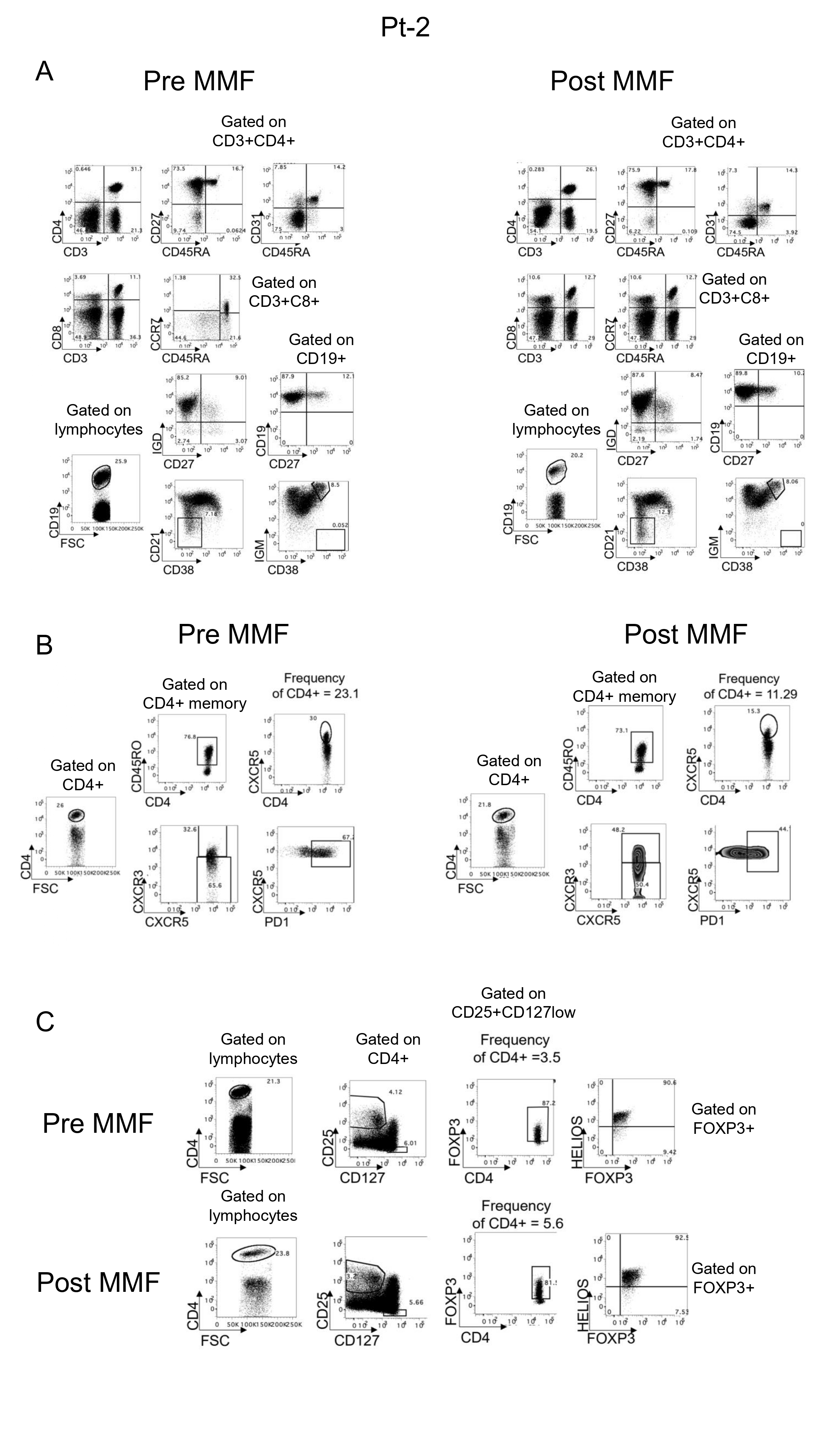

Supplement: Supplementary file 5 [file Image5.jpg]

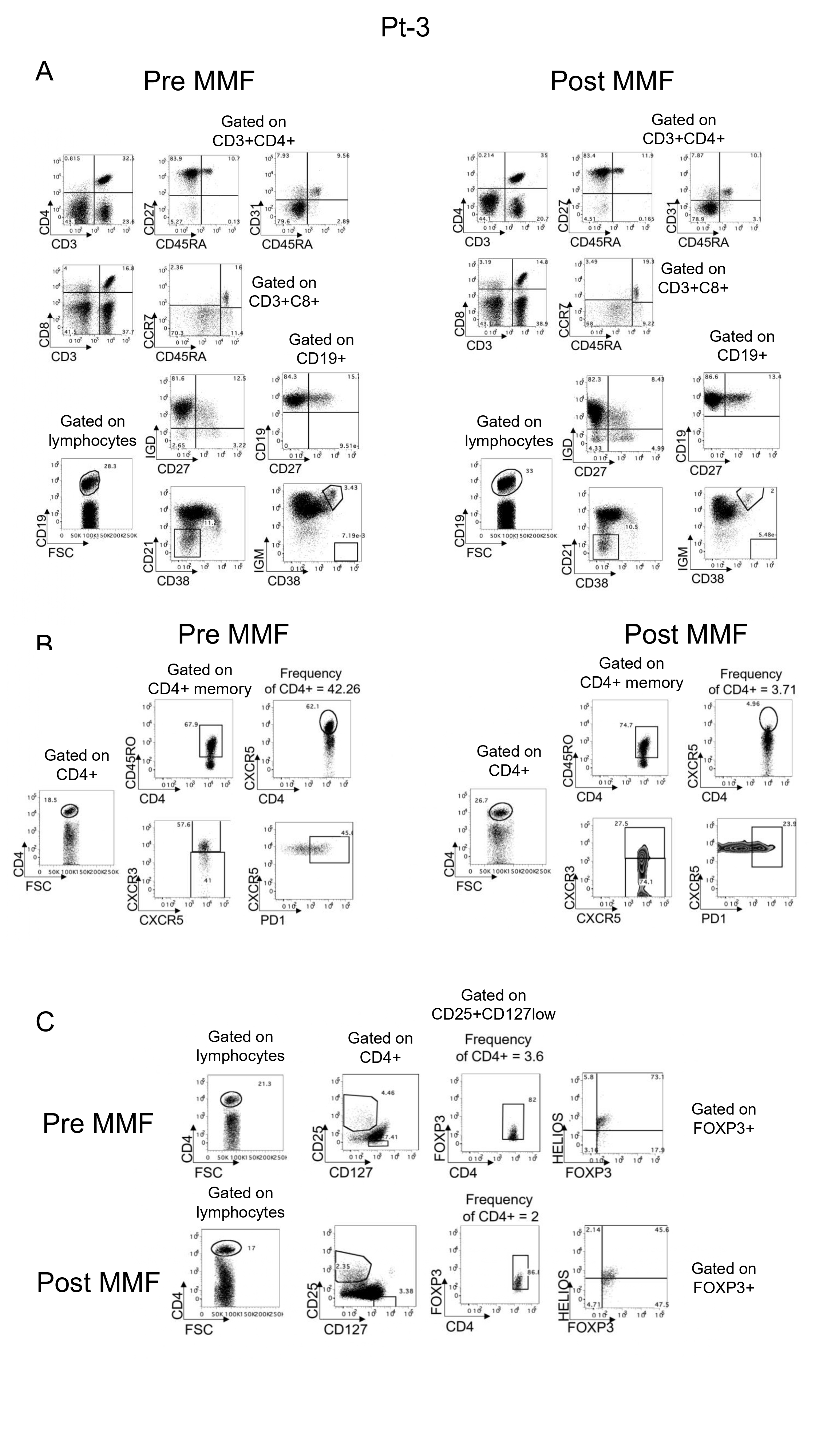

Supplement: Supplementary file 6 [file Image6.jpg]

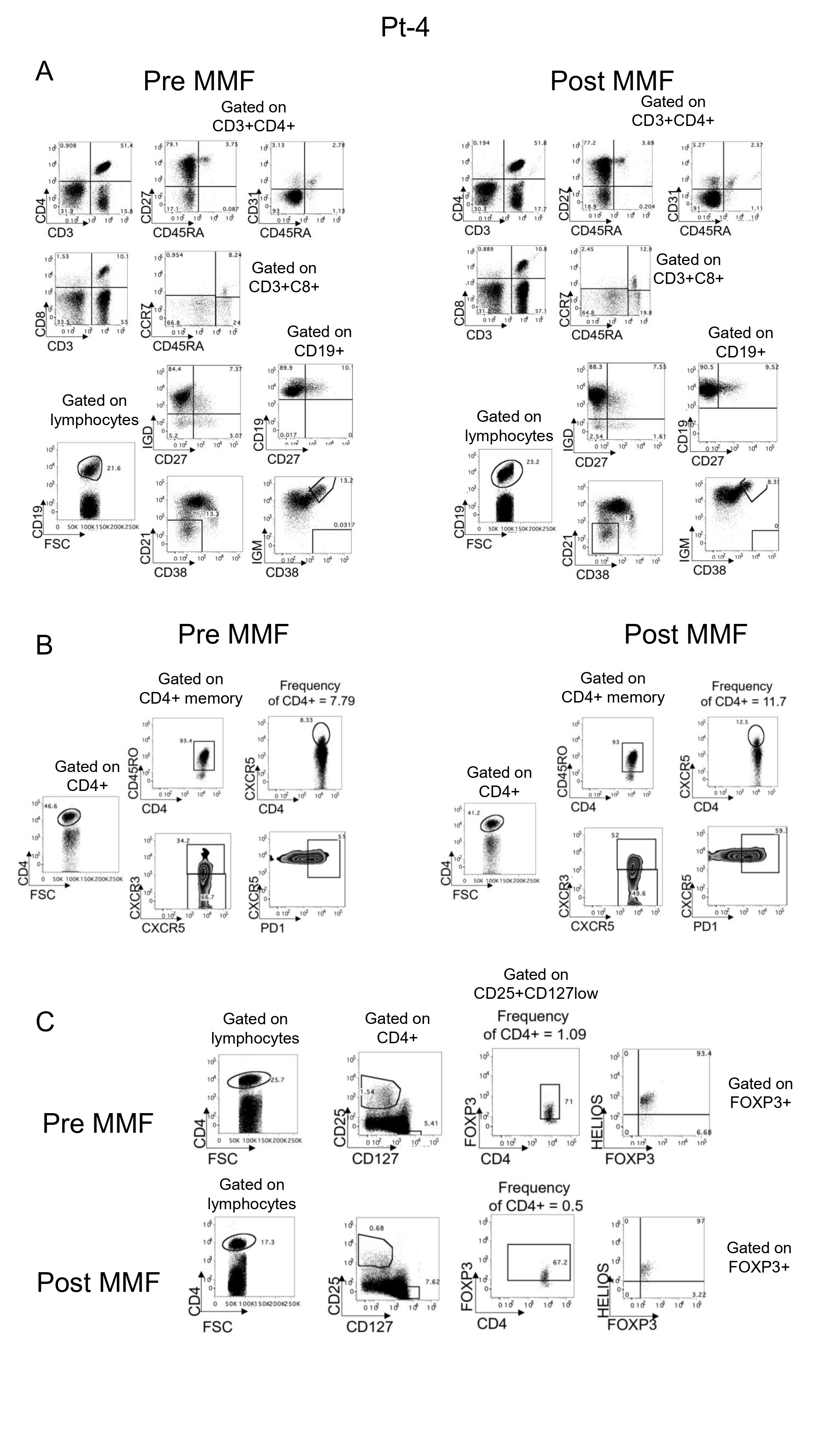

Supplement: Supplementary file 7 [file Image7.jpg]

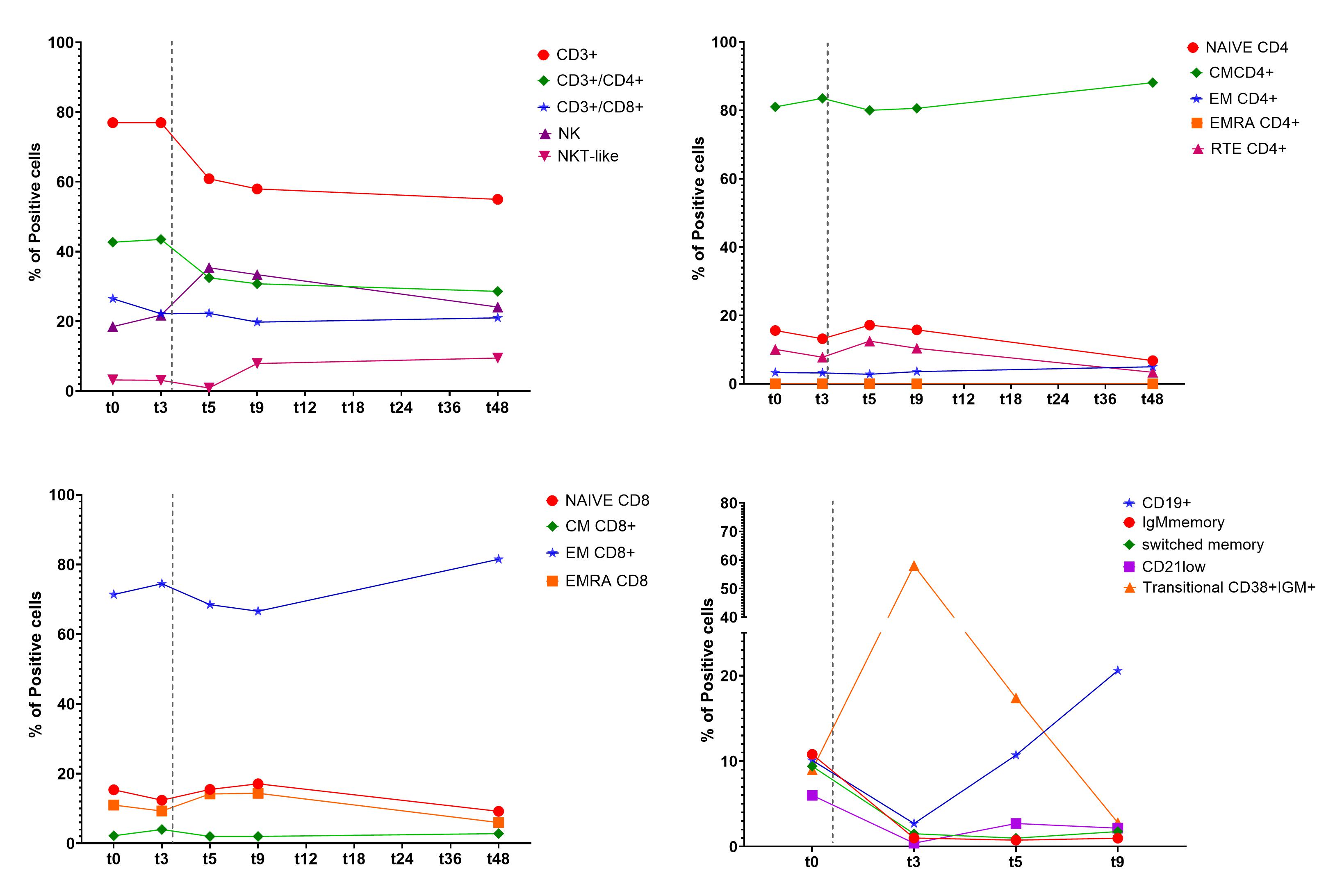

Supplement: Supplementary file 8 [file Image8.jpg]

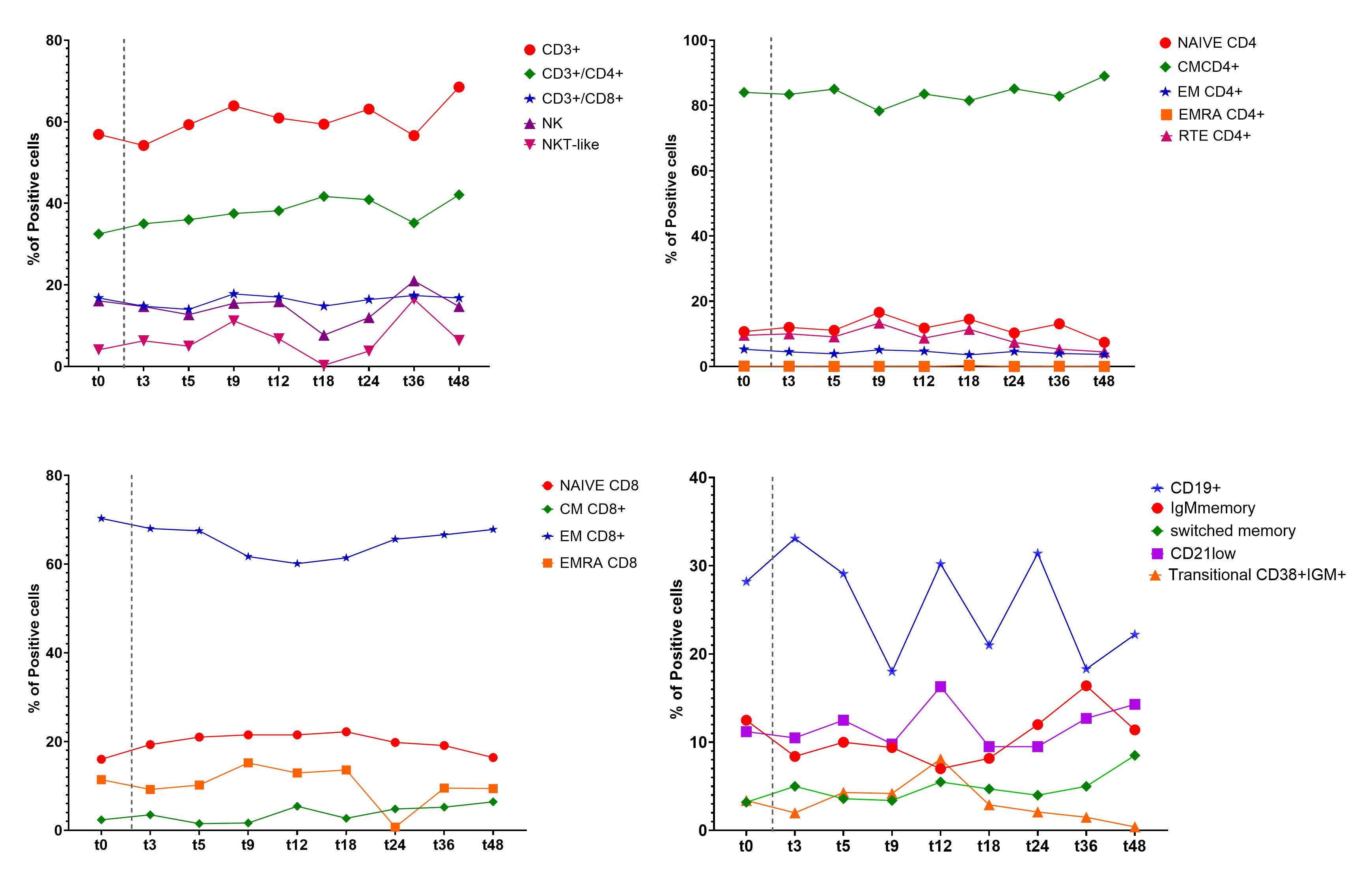

Supplement: Supplementary file 9 [file Image9.jpg]

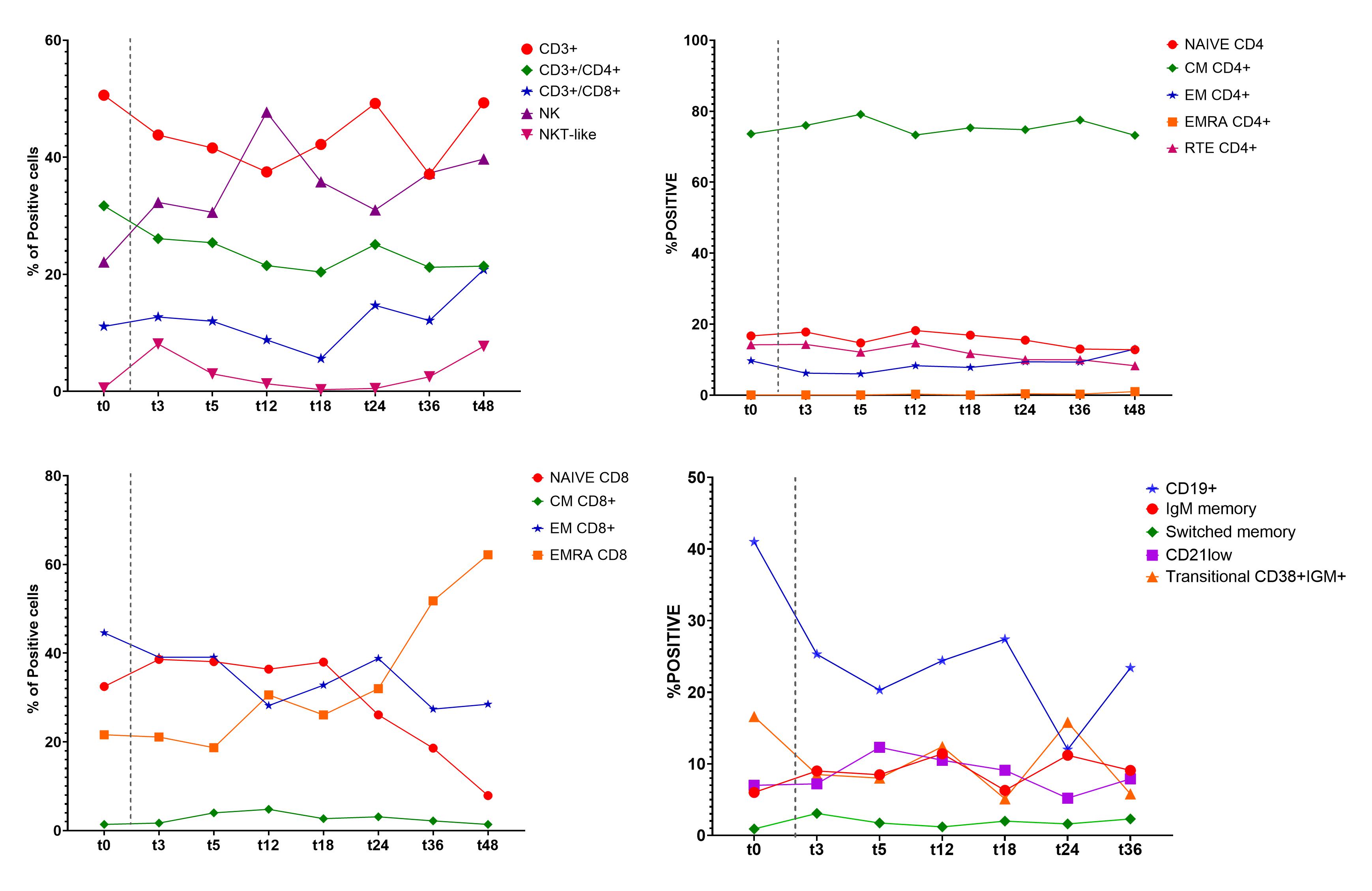

Supplement: Supplementary file 10 [file Image10.jpg]

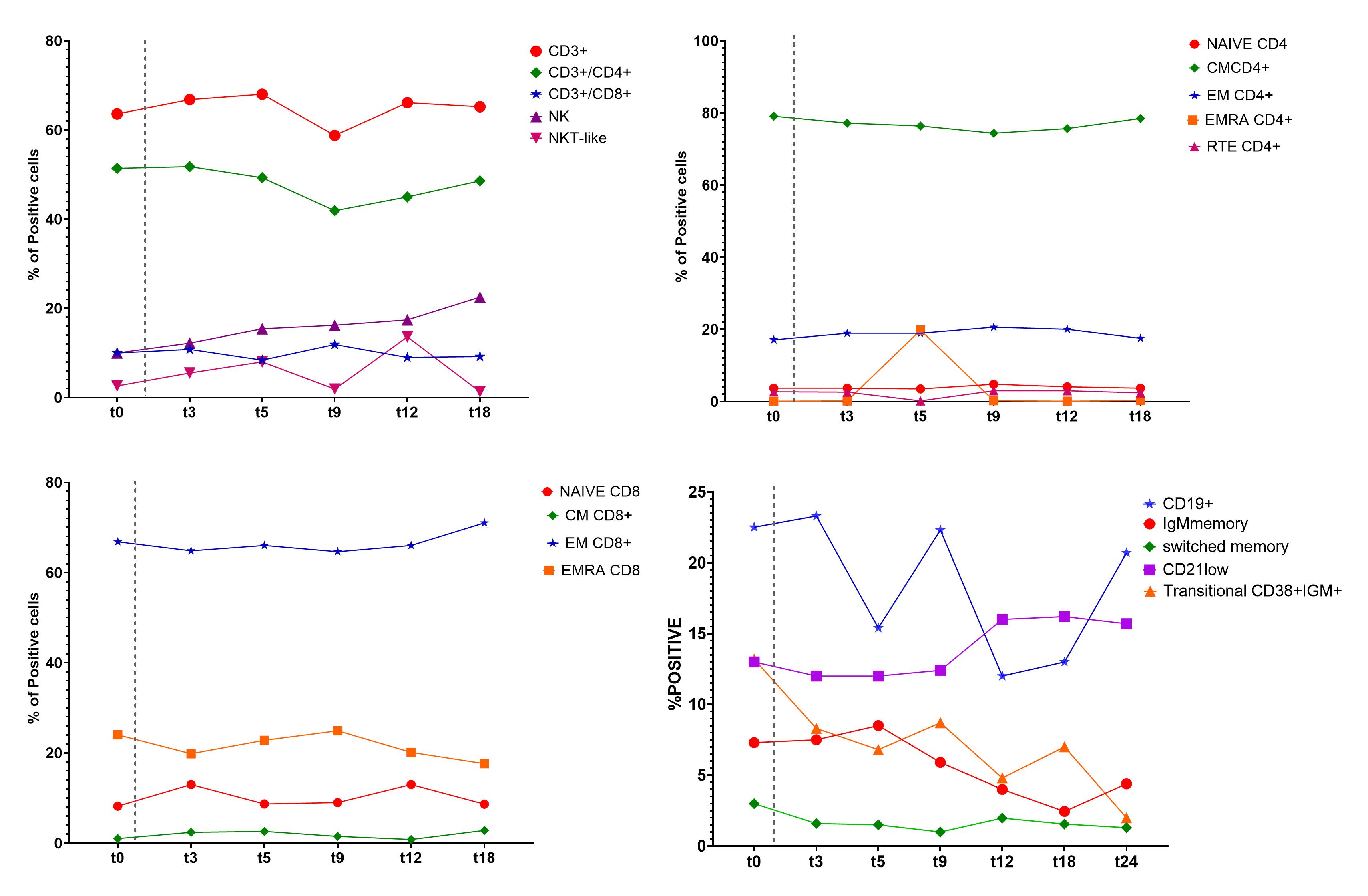

Supplement: Supplementary file 11 [file Image11.jpg]
