# Supplementary material for: Refractory immune cytopenia successfully treated with mycophenolate mofetil in four adolescents with del22q11.2 syndrome
Source: Front Immunol. 2026 May 13;17:1819182. doi: 10.3389/fimmu.2026.1819182 (PMC13212233; doi:10.3389/fimmu.2026.1819182)
Supplement: Supplementary file 12 [file SupplementaryFile1.docx]

**Supplementary figures legend**

**Supplementary Table 1. Definition of immune cell populations and corresponding marker combinations used for flow cytometry analyses.** The table summarizes the immune cell populations analyzed in this study together with the corresponding marker combinations used for their identification by multiparametric flow cytometry. Marker definitions were applied to characterize T-cell, B-cell, NK-cell, regulatory T-cell (Treg), and circulating T follicular helper (cTfh) compartments, including naïve, memory, effector, and atypical subsets.

**Supplementary Table 2. Mean serum immunoglobulin levels (IgG, IgA, IgM) before and after MMF treatment in patients with 22q11.2 deletion syndrome.** Mean serum immunoglobulin levels (IgG, IgA, IgM) before and after initiation of mycophenolate mofetil (MMF) therapy are shown for each patient. Immunoglobulin replacement therapy was administered in two patients (SCIg in Pt-1 and IVIg in Pt-4). Pre-MMF immunoglobulin values in Pt-1 were obtained before the initiation of subcutaneous immunoglobulin (SCIg) therapy. In contrast, pre-MMF immunoglobulin values in Pt-4 were measured during ongoing intravenous immunoglobulin (IVIg) replacement therapy and therefore reflect treated rather than baseline immunoglobulin levels Platelet response after MMF treatment is reported for each patient. Asterisks (*) indicate immunoglobulin values measured during ongoing IVIg replacement therapy.

**Supplementary figure 1. Gating strategy for identification of peripheral blood T-, B-, and NK-cell subsets. (A)** Representative gating strategy used to identify major lymphocyte populations from lysed whole blood samples. Lymphocytes were first selected based on forward scatter (FSC) and side scatter (SSC) properties and CD45 expression. T cells were identified as CD3⁺CD45⁺ lymphocytes, B cells as CD19⁺CD45⁺ cells, and NK cells as CD3⁻CD16⁺CD56⁺ cells. **(B)** Gating strategy for CD4⁺ T-cell subsets. CD3⁺CD4⁺ T cells were subdivided into naïve (N; CD45RA⁺CD27⁺), central memory (CM; CD45RA⁻CD27⁺), effector memory (EM; CD45RA⁻CD27⁻), and terminally differentiated effector memory RA⁺ (EMRA; CD45RA⁺CD27⁻) subsets. Recent thymic emigrants (RTE) were identified as CD45RA⁺CD31⁺ within the CD3⁺CD4⁺ compartment. **(C)** Gating strategy for CD8⁺ T-cell subsets. CD3⁺CD8⁺ T cells were classified into naïve (N; CD45RA⁺CCR7⁺), central memory (CM; CD45RA⁻CCR7⁺), effector memory (EM; CD45RA⁻CCR7⁻), and terminally differentiated effector memory RA⁺ (EMRA; CD45RA⁺CCR7⁻) populations.**(D)** Gating strategy for αβ+, γδ+ and double-negative (DN) TCR αβ⁺ T cells were defined as CD3⁺TCRαβ⁺CD4⁻CD8⁻ lymphocytes after exclusion of TCRγδ⁺ cells. Abbreviations: FSC, forward scatter; SSC, side scatter; NK, natural killer; N, naïve; CM, central memory; EM, effector memory; EMRA, terminally differentiated effector memory CD45RA⁺; RTE, recent thymic emigrants; DN, double negative.

**Supplementary Figure 2. Gating strategy for regulatory T cells (Tregs) and circulating T follicular helper (cTfh) cell subsets. (A)** Representative gating strategy for identification of regulatory T cells (Tregs). Lymphocytes were first gated based on FSC/SSC properties and CD4 expression. Tregs were defined as CD4⁺CD25⁺CD127^lowFOXP3⁺ cells. Within the FOXP3⁺ compartment, CD45RA expression was used to further characterize naïve and memory Treg subsets. Helios expression was analyzed within FOXP3⁺ cells as an additional marker associated with thymic-derived Tregs. **(B)** Gating strategy for circulating T follicular helper cells (cTfh). CD4⁺ memory T cells (CD45RA⁻) were selected, and cTfh cells were identified as CD4⁺CXCR5⁺ lymphocytes. cTfh subsets were further characterized based on CXCR3 expression to identify Tfh1-like (CXCR3⁺CXCR5⁺) and Tfh2-like (CXCR3⁻CXCR5⁺) populations. PD-1 expression was evaluated within the CXCR5⁺ compartment as a marker of activation. Abbreviations: Treg, regulatory T cell; cTfh, circulating T follicular helper cell; FOXP3, Forkhead box P3; PD-1, programmed cell death protein 1.

**Supplementary Figure 3. Gating strategy for peripheral B-cell subsets**. Representative gating strategy used to identify peripheral blood B-cell subsets from lysed whole blood samples. After selection of CD19⁺ lymphocytes, B cells were subdivided into naïve (CD27⁻IgD⁺), unswitched memory (CD27⁺IgD⁺), switched memory (CD27⁺IgD⁻), and double-negative (DN; CD27⁻IgD⁻) populations. CD21^lowCD38⁻ cells were identified as atypical memory B cells. Transitional B cells were defined as CD38⁺⁺IgM⁺⁺ cells within the CD19⁺ compartment, whereas plasmablasts were identified as CD38⁺⁺IgM⁻ cells. Abbreviations: DN, double negative.

**Supplementary Figure 4.** **Representative flow cytometry plots of** **longitudinal immunophenotypic changes before and after MMF treatment in pt.-1.** Representative flow cytometry plots illustrating peripheral blood lymphocyte subset distribution before initiation of mycophenolate mofetil (MMF) therapy (Pre-MMF) and during follow-up after treatment initiation (Post-MMF). (A) Representative plots showing CD4⁺ and CD8⁺ T-cell subsets (naïve, central memory, effector memory, and EMRA), recent thymic emigrants (RTE), and B-cell subsets including naïve, unswitched memory, switched memory, atypical memory (CD21^low), transitional B cells, and plasmablasts. (B) Representative plots showing circulating T follicular helper (cTfh) cells identified as CD4⁺CXCR5⁺ lymphocytes and their characterization according to CXCR3 expression (Tfh1-like) and PD-1 expression. (C) Representative plots showing regulatory T cells (Tregs) identified as CD4⁺CD25⁺CD127^lowFOXP3⁺ lymphocytes and further characterized by Helios.

**Supplementary Figure 5. Representative flow cytometry plots of longitudinal immunophenotypic changes before and after MMF treatment in pt-2.** Representative flow cytometry plots illustrating peripheral blood lymphocyte subset distribution before initiation of mycophenolate mofetil (MMF) therapy (Pre-MMF) and during follow-up after treatment initiation (Post-MMF). Post-treatment samples were obtained at approximately 3 months after MMF initiation for T- and B-cell subsets and circulating T follicular helper (cTfh) cells, and at approximately 9 months for regulatory T cells (Tregs) (A) Representative plots showing CD4⁺ and CD8⁺ T-cell subsets (naïve, central memory, effector memory, and EMRA), recent thymic emigrants (RTE), and B-cell subsets including naïve, unswitched memory, switched memory, atypical memory (CD21^low), transitional B cells, and plasmablasts. (B) Representative plots showing circulating T follicular helper (cTfh) cells identified as CD4⁺CXCR5⁺ lymphocytes and their characterization according to CXCR3 expression (Tfh1-like) and PD-1 expression. (C) Representative plots showing regulatory T cells (Tregs) identified as CD4⁺CD25⁺CD127^lowFOXP3⁺ lymphocytes and further characterized by Helios.

**Supplementary Figures 6. Representative flow cytometry plots of longitudinal immunophenotypic changes before and after MMF treatment in pt-3.** Representative flow cytometry plots illustrating peripheral blood lymphocyte subset distribution before initiation of mycophenolate mofetil (MMF) therapy (Pre-MMF) and during follow-up after treatment initiation (Post-MMF). (A) Representative plots showing CD4⁺ and CD8⁺ T-cell subsets (naïve, central memory, effector memory, and EMRA), recent thymic emigrants (RTE), and B-cell subsets including naïve, unswitched memory, switched memory, atypical memory (CD21^low), transitional B cells, and plasmablasts. (B) Representative plots showing circulating T follicular helper (cTfh) cells identified as CD4⁺CXCR5⁺ lymphocytes and their characterization according to CXCR3 expression (Tfh1-like) and PD-1 expression. (C) Representative plots showing regulatory T cells (Tregs) identified as CD4⁺CD25⁺CD127^lowFOXP3⁺ lymphocytes and further characterized by Helios.

**Supplementary Figure 7. Representative flow cytometry plots of longitudinal immunophenotypic changes before and after MMF treatment in pt-4.** Representative flow cytometry plots illustrating peripheral blood lymphocyte subset distribution before initiation of mycophenolate mofetil (MMF) therapy (Pre-MMF) and during follow-up after treatment initiation (Post-MMF). (A) Representative plots showing CD4⁺ and CD8⁺ T-cell subsets (naïve, central memory, effector memory, and EMRA), recent thymic emigrants (RTE), and B-cell subsets including naïve, unswitched memory, switched memory, atypical memory (CD21^low), transitional B cells, and plasmablasts. (B) Representative plots showing circulating T follicular helper (cTfh) cells identified as CD4⁺CXCR5⁺ lymphocytes and their characterization according to CXCR3 expression (Tfh1-like) and PD-1 expression. (C) Representative plots showing regulatory T cells (Tregs) identified as CD4⁺CD25⁺CD127^lowFOXP3⁺ lymphocytes and further characterized by Helios. **Supplementary Figure 8. Longitudinal immunophenotypic profiling of peripheral blood lymphocyte subsets before and after MMF treatment in pt-1.** Longitudinal evaluation of peripheral blood lymphocyte subsets in patient 1 (pt-1) at different timepoints before and after initiation of mycophenolate mofetil (MMF). The dashed vertical line indicates the start of MMF treatment. Data are expressed as a percentage of positive cells within the parent population.

**Supplementary Figure 9. Longitudinal immunophenotypic profiling of peripheral blood lymphocyte subsets before and after MMF treatment in pt-2.** Longitudinal evaluation of peripheral blood lymphocyte subsets in patient 1 (pt-1) at different timepoints before and after initiation of mycophenolate mofetil (MMF). The dashed vertical line indicates the start of MMF treatment. Data are expressed as a percentage of positive cells within the parent population.

**Supplementary Figure 10. Longitudinal immunophenotypic profiling of peripheral blood lymphocyte subsets before and after MMF treatment in pt-3.** Longitudinal evaluation of peripheral blood lymphocyte subsets in patient 1 (pt-1) at different timepoints before and after initiation of mycophenolate mofetil (MMF). The dashed vertical line indicates the start of MMF treatment. Data are expressed as a percentage of positive cells within the parent population.

**Supplementary Figure 11. Longitudinal immunophenotypic profiling of peripheral blood lymphocyte subsets before and after MMF treatment in pt-4.** Longitudinal evaluation of peripheral blood lymphocyte subsets in patient 1 (pt-1) at different timepoints before and after initiation of mycophenolate mofetil (MMF). The dashed vertical line indicates the start of MMF treatment. Data are expressed as a percentage of positive cells within the parent population.
