# Supplementary material for: Refractory immune cytopenia successfully treated with mycophenolate mofetil in four adolescents with del22q11.2 syndrome
Source: Front Immunol. 2026 May 13;17:1819182. doi: 10.3389/fimmu.2026.1819182 (PMC13212233; doi:10.3389/fimmu.2026.1819182)
Supplement: Supplementary file 13 [file Table1.docx]

**Supplementary Table 1. Definition of immune cell populations and corresponding marker combinations used for flow cytometry analyses.**

| Cell population | Marker |
| --- | --- |
| T lymphocytes | CD3+CD45+ |
| CD4+ T lymphocytes | CD3+CD4+ |
| CD4+ Naive T lymphocytes (T_N_) | CD3+CD4+CD27+CD45RA+ |
| CD4+ Central memory T lymphocytes (T_CM_) | CD3+CD4+CD27+CD45RA- |
| CD4+ Effector Memory T lymphocytes (T_EM_) | CD3+CD4+CD27-CD45RA- |
| CD4+ Effector Memory cells re-expressing CD45RA (T_EMRA_) | CD3+CD4+CD27-CD45RA+ |
| CD4+ Recent thymic emigrants T lymphocytes (RTE) | CD3+CD4+CD31+CD45RA+ |
| CD8+ T lymphocytes | CD3+CD8+ |
| CD8+ Naive T lymphocytes (T_N_) | CD3+CD8+CCR7+CD45RA+ |
| CD8+ Central memory T lymphocytes (T_CM_) | CD3+CD8+CCR7+CD45RA- |
| CD8+ Effector Memory T lymphocytes (T_EM_) | CD3+CD8+CCR7-CD45RA- |
| CD8+ Effector Memory cells re-expressing CD45RA (T_EMRA_) | CD3+CD8+CCR7-CD45RA+ |
| CD3+ T cell Receptor (TCR) αβ+ | CD3+TCRαβ |
| CD3+ T cell Receptor (TCR) γδ+ | CD3+TCRγδ |
| Double negative (DN) TCRαβ+ T cells | CD3+CD4-CD8-TCRαβ+ |
| Regulatory T cells | CD4+CD25hiCD127lowFoxp3+ |
| Circulating follicular T helper cells | CD4+CD45RA-CXCR5+ |
| Circulating follicular T helper 1-like cells (Th1-like) | CD4+CD45RA-(or CD45RO+) CXCR5+CXCR3+ |
| Circulating follicular T helper 2-like cells (Th2-like) | CD4+-CD45RA-(or CD45RO+) CXCR5+CXCR3- |
| B lymphocytes | CD19+CD45+ |
| CD19+ Unswitched Memory B lymphocytes | CD27+IgD+IgM+ |
| CD19+ Switched Memory B lymphocytes | CD27+IgD-IgM- |
| CD19+ Naïve B lymphocytes | CD27-IgD+IgM+ |
| CD19+ Double Negative B lymphocytes | CD27-IgD-IgM- |
| CD19+ Atypical Memory B lymphocytes | CD21low CD38low |
| CD19+ Transitional B lymphocytes | IgMhigh CD38high |
| CD19+ PlasmaBlast B lymphocytes | CD38++IgM- |
| NK cells | CD3-CD16+CD56+ |
