# Supplementary material for: Refractory immune cytopenia successfully treated with mycophenolate mofetil in four adolescents with del22q11.2 syndrome
Source: Front Immunol. 2026 May 13;17:1819182. doi: 10.3389/fimmu.2026.1819182 (PMC13212233; doi:10.3389/fimmu.2026.1819182)
Supplement: Supplementary file 14 [file Table2.docx]

| **Patient** | **Ig replacement** | **IgG mean (g/L)  Pre-MMF** | **IgA mean (g/L) Pre-MMF** | **IgM mean (g/L) Pre-MMF** | **IgG mean (g/L)  Post-MMF** | **IgA mean (g/L) Post-MMF** | **IgM mean (g/L) Post-MMF** | **Platelet response after MMF** | **Notes** |
| --- | --- | --- | --- | --- | --- | --- | --- | --- | --- |
| Pt-1 | SCIg | 16,78 | 1,21 | 0,9 | 16,82 | 1,47 | 0,73 | Complete response | Ig used as supportive therapy |
| Pt-2 | No | 10,44 | 0,74 | 0,69 | 5,53 | 0,62 | 0,72 | Complete response | — |
| Pt-3 | No | 9,70 | 0,50 | 0,69 | 8,98 | 0,44 | 0,75 | Complete response | — |
| Pt-4 | IVIg | 10,04* | 0,38* | 1,02* | 7,80 | 0,27 | 0,33 | Partial response | Low adherence |

**Table 2. Mean serum immunoglobulin levels before and after MMF treatment**
